# COVID-19 National Football League (NFL) Injury Analysis: Follow-Up Study

**DOI:** 10.2196/45688

**Published:** 2024-02-13

**Authors:** Troy B Puga, Joshua Schafer, Grace Thiel, Nicholas Scigliano, Tiffany Ruan, Andres Toledo, Prince N Agbedanu, Kevin Treffer

**Affiliations:** 1 College of Osteopathic Medicine, Kansas City University, Kansas City, MO, United States; 2 School of Medicine, University of Kansas, Kansas City, KS, United States; 3 Division of Science, Technology, Engineering, and Math, Department of Health Sciences, Friends University, Wichita, KS, United States; 4 Department of Osteopathic Manipulative Medicine, College of Osteopathic Medicine, Kansas City University, Kansas City, MO, United States

**Keywords:** COVID-19, injury, prevalence, adaptation, sports medicine, follow-up, training, football, epidemiology, sport, athlete, athletic, injuries

## Abstract

**Background:**

In 2020, COVID-19 spread across the world and brought normal daily life to a halt, causing the shutdown of nearly everything in order to prevent its spread. The National Football League (NFL) similarly experienced shutdowns and the resulting effects, leaving athletes unable to train in some of the most advanced facilities with many of the best trainers in the world. A previous study, titled *COVID-19 Return to Sport: NFL Injury Prevalence Analysis*, determined that there was increased injury prevalence during the 2020 season, likely due to decreased physiological adaptations within athletes’ bodies as a result of facility shutdowns. Understanding injury epidemiology is vital to the prevention of injuries and the development of return-to-play protocols.

**Objective:**

The objective of this study is to perform a follow-up study to *COVID-19 Return to Sport: NFL Injury Prevalence Analysis* in order to examine the longitudinal effects of the COVID-19 pandemic on injury epidemiology. This study examines if there was a recovery to baseline levels of injuries or if there are still lingering effects from the COVID-19 pandemic–induced spike in injuries.

**Methods:**

To determine if there was change in the number of injuries for each season, injury tallies collected from the 17-week-long 2018, 2019, and 2020 NFL regular seasons were compared with those from the 18-week-long 2021 and 2022 NFL regular seasons. A Kruskall-Wallis test with post hoc Dunn analysis was conducted to compare the rate of injuries per team per week between each of the 2018, 2019, 2020, 2021, and 2022 regular seasons.

**Results:**

The Kruskall-Wallis test revealed an *H* statistic of 32.61 (*P*<.001) for the comparison of the injury rates across the 5 seasons. The post hoc Dunn analysis showed that 2020 had a statistically significant difference when compared with each of the 2018 (*P*<.001), 2019 (*P*=.04), 2021 (*P*=.02), and 2022 (*P*=.048) seasons. The 2019 season showed no statistical significance when compared with the 2021 (*P*=.23) and 2022 (*P*=.13) seasons.

**Conclusions:**

The results of this follow-up study, combined with the previous study, show that extended training interruptions stemming from COVID-19 in 2020 induced detraining and led to increased injuries. Additionally, the results of this study show that retraining can occur, resulting in the development of injury protective factors, as injury rates returned to baseline levels after 2020. This is the first large-scale and long-term opportunity to demonstrate the effects of these principles and how they are important to understanding injury epidemiology.

## Introduction

In 2020, the COVID-19 pandemic spread across the world and led to the shutdown of everything except essential services. Sports were no exception, as competition came to an immediate halt. The National Football League (NFL) was one such sport that was shutdown, as they closed all facilities from late March 2020 until late May 2020 [1,2]. While facilities opened back up in May, players were unable to return until the end of July [3]. This shutdown eliminated most of the training period, giving players a narrow 20-day period to reacclimate before the start of the 2020 season [3]. A previous study, titled *COVID-19 Return to Sport: NFL Injury Prevalence Analysis,* examined the effects of this shutdown on injury epidemiology [2]. This study showed that there was increased injury prevalence during the 2020 NFL season, which was impacted by the COVID-19 pandemic [2]. The study concluded that the increased injury prevalence was due to a decline in athlete training as a result of the shutdowns [2]. The decline in training, and hence, the lack of bodily preparation for the 2020 NFL season, resulted in decreased physiological adaptations of players’ bodies to strenuous exertions, which is a hallmark of the sport [2]. This study serves as a follow-up study to examine the longitudinal effects of the COVID-19 pandemic shutdowns on injury prevalence in the NFL following the 2020 NFL season.

The NFL is an American football league that is composed of 32 teams with some of the best athletes in the world. The NFL is widely recognized as having some of the best trainers and facilities to ensure that these high-level athletes are prepared to play each season. NFL athletes follow intense training in order to physically prepare their bodies for the demand of a strenuous season [4]. Although training may not prevent every injury, research has shown that training produces a protective effect from injury [5-7]. Traditionally, the NFL was played over a 17-week-long NFL season each year until the 2021 season, when they transitioned to an 18-week-long NFL season for all future seasons [8].

Previous research demonstrated that the COVID-19 pandemic had acute effects on injury prevalence during the 2020 NFL season [2]. Much of this can be attributed to the decreased physiological adaptations that occurred during shutdowns, when athletes were unable to access NFL training facilities [1,2]. It is important to note that although teams could provide players with up to US $1500 worth of at-home training equipment, the players still lacked access to on-site athletic trainers and recovery facilities and were unable to partake in normal preseason training [9]. Any at-home workouts were considered voluntary [9]. Therefore, these athletes most likely exhibited the effects of detraining, a process in which athletes undergo a loss of physiological and performance-based adaptations from previous training due to a lack of sufficient training [3,10]. It is believed that many athletes across the United States and the world exhibited the effects of acute detraining during the COVID-19 shutdown [3,11]. Previous shutdowns in the NFL have shown that detraining can occur, such as during the 2011 lockout, where the preseason saw an increased number of Achilles tendon injuries [12]. Training is one of the most important interventions for injury prevention and improved athletic performance in athletes of all populations [5-7,13-15].

The effects of detraining are a serious concern in regard to injury epidemiology. We can clearly see that detraining has significant potential to lead to injuries; however, complete recovery from detraining can be obtained through a sufficient training program [3,16]. The amount of time necessary to recover from the effects of detraining is widely debated [3]. The amount of time needed for training adaptations often differs between people based on training levels, genetics, and a host of other factors [3,17-19]. Many of these genetic and environmental factors contribute to the variability of injury recovery times and why some athletes may recover more quickly [20-22]. Although we know that recovery is complex and difficult to predict, research has suggested that the time necessary to fully return from detraining is often similar to or longer than the length of detraining [16]. The variations in training adaptations and recovery from detraining makes the examination of the longitudinal effects of the COVID-19 pandemic that much more important to understanding longitudinal injury epidemiology.

Longitudinal research on injury epidemiology due to a major event, such as the COVID-19 pandemic, is scarce. The goal of this research is to understand the effects of the COVID-19 pandemic on longitudinal injury epidemiology. The authors hypothesized that injury prevalence for the 2021 and 2022 NFL seasons would be lower than that of the 2020 NFL season, which was affected by the COVID-19 pandemic. Correspondingly, it was hypothesized that decreased injury prevalence would result from athletes having full access to sports performance facilities and staff to properly train their bodies during the 2021 and 2022 NFL seasons. It is believed that during these 2 seasons, athletes had adequate time and preparation to induce the necessary physiological changes and undergo retraining, thus avoiding the effects of detraining observed following the COVID-19 pandemic–induced shutdowns.

## Methods

### Ethical Considerations

This study did not require institutional review board approval as all data are publicly available and this study does not qualify as human subjects research per US Department of Health and Human Services policy 45 CFR 46.102.f.

### Study Design

The methodology for this study design was adapted from the previous study, *COVID-19 Return to Sport: NFL Injury Prevalence Analysis* [2]. Data for the 2020 NFL regular season were collected in the previous study and used for further analysis [2]. The number of injuries was tallied for the 18-week-long 2021 and 2022 NFL regular seasons using the weekly published injury reports by each NFL team. Injury reports are made publicly available by each team in the NFL. If an official injury report was not available through the individual team media, a deferment was made to the official NFL website. Per the NFL injury report policy, teams are required to report injuries throughout the week of a game, so any and all information should be considered accurate [23]. Athletes listed with the same injury for consecutive weeks were only counted once in order to prevent any repeat data. If an athlete presented with an injury to a different anatomical region, this was counted as a new unique injury. No data were repeated at any point during the collection process. Contact injuries were included in this study as this is a nonmodifiable risk factor that cannot be controlled due to football being a contact sport [2,24]. COVID-19 infection, sick days, and nonmedical days off were not included in the injury tally. Illnesses were not included in this study, because illnesses are not considered a physical injury and should be reported separately from injuries when performing injury epidemiological studies [2,25]. All other soft tissue injuries and concussions were included during the first week of the associated injury report. The 2020 season, which was played after the COVID-19–induced shutdowns, was used for comparison with the subsequent 2021 and 2022 seasons.

### Data Analysis

The data analysis for this study was conducted differently from the previous study [2], as there is a need to correct for the change from the 17-week-long NFL seasons in 2018, 2019, and 2022 to the 18-week-long NFL seasons in 2021 and 2022 [8]. The comparison required first dividing the number of injuries per team by the number of weeks for each season. This adjustment allowed for the comparison of 2 different regular season lengths. A Kruskall-Wallis test with post hoc Dunn analysis was conducted on the number of injuries per team per week rate to compare the 2018, 2019, 2020, 2021, and 2022 seasons.

## Results

**Figure 1.**
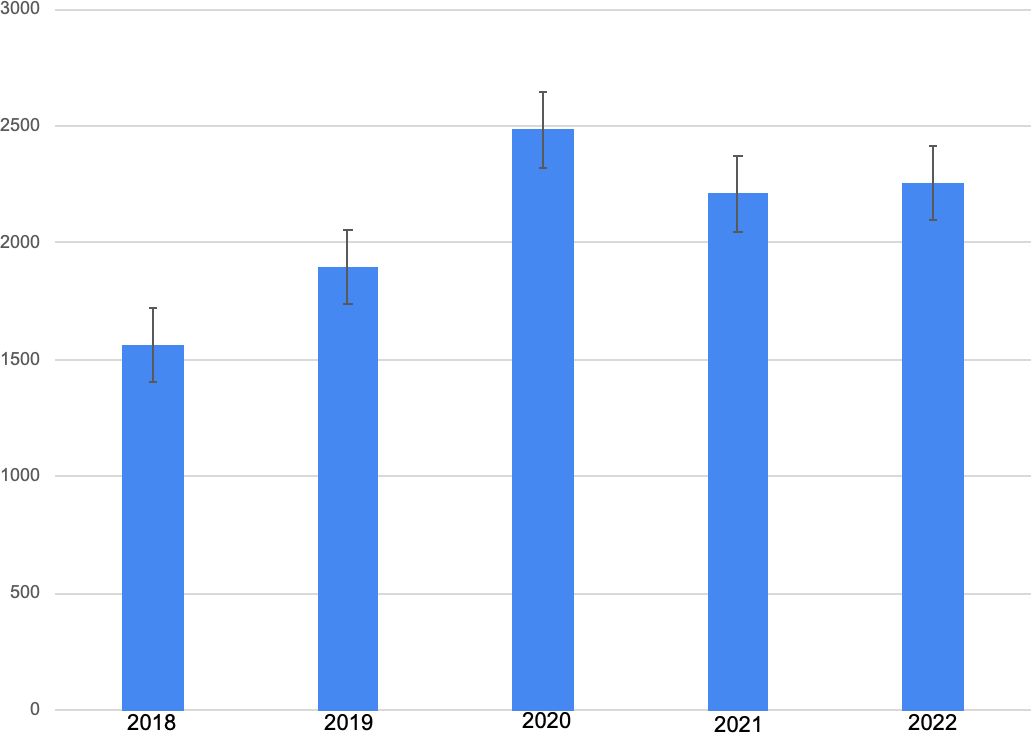
The total number of injuries per NFL regular season, including the 17-week-long 2018, 2019, and 2020 NFL seasons from the previous study [2] and the 18-week-long 2021 and 2022 NFL seasons. Error bars signify SE. NFL: National Football League.

The 2018 season had a total of 1561 injuries (Figures1  2) [2]. This number was divided by 17, the number of weeks in the NFL regular season for the 2018, 2019, and 2020 seasons, to produce a rate that allowed for an appropriate comparison with the change to 18-week seasons in 2021 and 2022. Thus, the 2018 season produced a rate of 91.8 injuries per week and a rate of 2.9 injuries per team per week. The 2019 season had a total of 1897 injuries [2], producing a league-wide rate of 111.6 injuries per week and a rate of 3.5 injuries per team per week. The 2020 season had a total of 2484 injuries, as indicated in the previous study [2]. It produced a league-wide rate of 146.1 injuries per week and a rate of 4.6 injuries per team per week. The 2021 season had a total of 2210 injuries, which was divided by 18 to correspond with the number of weeks in the 2021 regular season. This produced a league-wide rate of 122.8 injuries per week and a rate of 3.8 injuries per team per week. The 2022 season had a total of 2257 injuries. This was also divided by 18 to represent the number of weeks in the 2022 regular season. This produced a league-wide rate of 125.4 injuries per week and a rate of 3.3 injuries per team per week.

Data analysis with a Kruskall-Wallis test found that the rate of injuries per team per week differed between the 5 seasons with an *H* statistic of 32.61 (*P<*.001). From the post hoc Dunn analysis, the rate of injuries per team per week for the 2018 NFL season was statistically significantly different than those of the 2019 (*P*=.04), 2020 (*P*<.001), 2021 (*P*<.001), and 2022 (*P*<.001) seasons. The rate of injuries per team per week for the 2019 NFL season was statistically significantly different than that of the 2020 (*P=.*04) season, but there was no significant difference when compared with those of the 2021 (*P=*.23) and 2022 (*P=.*13) seasons. The rate of injuries per team per week for the 2020 COVID-19–impacted NFL season was statistically significantly different than those of the 2021 (*P*=.02) and 2022 (*P*=.048) seasons. Comparison of the rate of injuries per team per week of the 2021 and 2022 seasons did not produce a statistically significant difference (*P*=.76).

**Figure 2.**
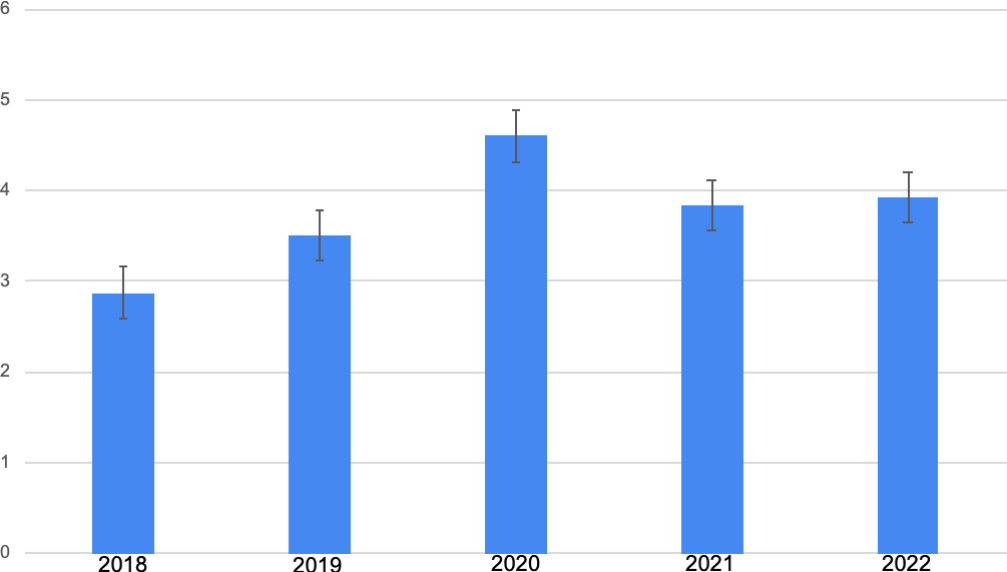
Number of injuries per week per team for each NFL regular season from 2018‐2022. Error bars signify SE. NFL: National Football League.

## Discussion

The study results showed statistically significant differences when comparing the 2020 COVID-19–impacted NFL season with the previous 2018 and 2019 seasons, as well as when compared with the subsequent 2021 and 2022 NFL seasons. The study also showed that there was a lower total number of injuries across the league in 2021 (2210 injuries) and 2022 (2257 injuries) when compared with the 2020 (2484 injuries) COVID-19–impacted NFL season. This occurred even with the addition of an extra week of regular season play in the 2021 and 2022 seasons when compared with the 2020 season [8]. The 2021 and 2022 seasons showed no statistically significant difference when compared with the 2019 season, indicating that injury levels returned to a similar level after 2020. This shows that there was a clear epidemiological spike in 2020. The previous study showed the impact of the COVID-19 pandemic, which led to a higher number of injuries in the 2020 NFL season when compared with earlier seasons—likely due to decreased training adaptations. This was thought to have stemmed from facility lockdowns aimed at mitigating the spread of COVID-19 [2]. This study demonstrated recovery from that spike back to prepandemic season levels, indicating that retraining and recovery from detraining is possible.

Previous research has demonstrated the effects of shutdowns and detraining, which lead to a negative impact on athletic performance and health [2,3,10-12]. Preparation for athletic competition is vital for the health and performance of athletes [5-7,13-15]. This follow-up study aimed to examine if the COVID-19 pandemic’s effects on injury epidemiology persisted or if athletes were able to return their bodies back to peak prepandemic shape. The decline in injuries after the COVID-19 pandemic season, as demonstrated in this follow-up study, showed that athletes were able to return to prepandemic athletic conditioning. This was most likely achieved through having a full offseason with full-access training for the 2021 and 2022 seasons. NFL players train in some of the best facilities in the world while under the supervision of renowned sports medicine personnel. It can be concluded from this follow-up study that the COVID-19 pandemic did have an acute effect on injuries, with a spike in the 2020 season. However, the COVID-19 pandemic did not have a longitudinal effect on injuries, as demonstrated by the decrease in injuries during the 2021 and 2022 regular seasons when compared with 2020. This follow-up study further suggests that athletic preparation through performance training is vital for the preparation of sport and injury prevention.

However, this study is not without limitations, and these limitations are consistent with the previous study. Similar to the previous study, this study is limited by the potential underreporting of injuries by players, unbeknownst to team personnel [2]. Another potential limitation, consistent with the previous study, is that the exact difference in training hours between seasons is unknown and variable by team to some degree [2]. However, it is known that there were limitations for training in 2020 [1] and that any survey administered about training limitations would be limited due to recall bias [2]. It is also possible that other factors influenced the findings of this study. However, the span of years included would hopefully account for any other possible differences besides the COVID-19–induced lockdown. The authors believe that despite these limitations, this study provides an accurate representation of the effects of detraining on injury epidemiology.

This research has demonstrated the importance of performance training for sport on injury epidemiology. This study shows that recovery from detraining is possible under the proper training conditions, in which athletes induce adaptation and preparation for sport. This is the first large-scale opportunity available to study detraining and retraining principles from a long-term perspective. Findings from this study serve as a foundational piece of research in proving what many have been hypothesized in regard to training and injury prevention.

Further studies must determine exactly how much time is needed to return to sport from a major event such as the COVID-19 pandemic, injuries, or time off. Qualified performance coaches and rehabilitation professionals are vital to help solve these issues. Further research must also be done at the collegiate and amateur levels to examine the effects of detraining and retraining, as these individuals may not have access to the care and facilities that NFL players receive. The findings from the previous study and this follow-up study serve as a starting point to future discussions of the necessary preparation times and levels. Further injury epidemiological trials and observational studies must be conducted to continue the evolution of return-to-sport protocols and training programs to combat detraining in adverse situations.

## Abbreviations

NFL: National Football League

## References

[1] Barrabi T (2020). NFL facilities can reopen from coronavirus shutdown starting May 19, Goodell says. Fox Business.

[2] Puga TB, Schafer J, Agbedanu PN, Treffer K (2022). COVID-19 return to sport: NFL injury prevalence analysis. JMIRx Med.

[3] Girardi M, Casolo A, Nuccio S, Gattoni C, Capelli C (2020). Detraining effects prevention: a new rising challenge for athletes. Front Physiol.

[4] Ebben WP, Blackard DO (2001). Strength and conditioning practices of National Football League strength and conditioning coaches. J Strength Cond Res.

[5] Gabbett TJ (2016). The training-injury prevention paradox: should athletes be training smarter and harder?. Br J Sports Med.

[6] Lauersen JB, Bertelsen DM, Andersen LB (2014). The effectiveness of exercise interventions to prevent sports injuries: a systematic review and meta-analysis of randomised controlled trials. Br J Sports Med.

[7] Lauersen JB, Andersen TE, Andersen LB (2018). Strength training as superior, dose-dependent and safe prevention of acute and overuse sports injuries: a systematic review, qualitative analysis and meta-analysis. Br J Sports Med.

[8] Graziano D (2021). NFL moves to 17-game regular season in 2021: what it means for teams, players, revenue, and fans. ESPN.

[9] Battista J (2020). Virtual offseason program set to begin April 20. NFL.

[10] Mujika I, Padilla S (2000). Detraining: loss of training-induced physiological and performance adaptations. part I: short term insufficient training stimulus. Sports Med.

[11] Nakisa N, Rahbardar MG (2021). Evaluating the probable effects of the COVID-19 epidemic detraining on athletes’ physiological traits and performance. Apunts Sports Medicine.

[12] Myer GD, Faigenbaum AD, Cherny CE, Heidt RS Jr, Hewett TE (2011). Did the NFL lockout expose the Achilles heel of competitive sports?. J Orthop Sports Phys Ther.

[13] Harries SK, Lubans DR, Callister R (2012). Resistance training to improve power and sports performance in adolescent athletes: a systematic review and meta-analysis. J Sci Med Sport.

[14] Kraemer WJ, Ratamess NA, French DN (2002). Resistance training for health and performance. Curr Sports Med Rep.

[15] Øvretveit K, Tøien T (2018). Maximal strength training improves strength performance in grapplers. J Strength Cond Res.

[16] Joo CH (2018). The effects of short term detraining and retraining on physical fitness in elite soccer players. PLoS One.

[17] Vikmoen O, Raastad T, Ellefsen S, Rønnestad BR (2020). Adaptations to strength training differ between endurance-trained and untrained women. Eur J Appl Physiol.

[18] Bouchard C, Rankinen T, Timmons JA (2011). Genomics and genetics in the biology of adaptation to exercise. Compr Physiol.

[19] Mann TN, Lamberts RP, Lambert MI (2014). High responders and low responders: factors associated with individual variation in response to standardized training. Sports Med.

[20] Lim T, Santiago C, Pareja-Galeano H (2021). Genetic variations associated with non-contact muscle injuries in sport: a systematic review. Scand J Med Sci Sports.

[21] Booth-Kewley S, Schmied EA, Highfill-McRoy RM, Sander TC, Blivin SJ, Garland CF (2014). A prospective study of factors affecting recovery from musculoskeletal injuries. J Occup Rehabil.

[22] Kvist J (2004). Rehabilitation following anterior cruciate ligament injury: current recommendations for sports participation. Sports Med.

[23] (2017). 2017 personel (injury) report policy. NFL.

[24] Emery CA (2005). Injury prevention and future research. Med Sport Sci.

[25] Fuller CW, Ekstrand J, Junge A (2006). Consensus statement on injury definitions and data collection procedures in studies of football (soccer) injuries. Clin J Sport Med.

